# Improving physical quality of life with group physical activity in the adjunctive treatment of major depressive disorder

**DOI:** 10.1186/1745-0179-4-1

**Published:** 2008-01-26

**Authors:** Mauro Giovanni Carta, Maria Carolina Hardoy, Alessandra Pilu, Manlio Sorba, Anna Laura Floris, Francesca Ada Mannu, Antonia Baum, Alessandra Cappai, Claudio Velluti, Massimiliano Salvi

**Affiliations:** 1 Department of Public Health, University of Cagliari, Cagliari, Italy and University Center for Research and Clinical Practice in Mental Health, University of Cagliari (Cagliari) and ASL 7 Iglesias (Iglesias), Italy; 2 Orthopaedic Clinic, University of Cagliari, Ospedale Marino, Cagliari, Italy; 3 Italian Society of Sport Psychiatry, Viale Merello 22, 09123 Cagliari, Italy; 4 Department of Psychiatry, Fairfax Hospital, 3300 Gallows Road, Falls Church, VA 22046, USA

## Abstract

**Background:**

The aim of the study was to compare the change in quality of life over 32 weeks in depressed women assuming antidepressant drug with (experimental group) or without (control group) physical exercise from a study which results on objective dimension of outcome were already published.

**Methods:**

Trial with randomized naturalistic control. Patients selected from the clinical activity registries of a Psychiatric University Unit. Inclusion criteria: female, between 40 and 60 years, diagnosis of Major Depressive Disorders (MMD, DSM-IV TR) resistant to ongoing treatment. Exclusion criteria: diagnosis of psychotic disorders; any contraindications to physical activity. 30 patients (71.4% of the eligible) participated to the study. Cases: 10 randomized patients undergoing pharmacological treatment plus physical activity. Controls: 20 patients undergoing only pharmacological therapy. Quality of life was measured by means of WHOQOL-Bref.

**Results:**

The patients that made physical activity had their WHOQOL-Bref physical score improved from T0 to T8, the differences was statistically significant. In the control group WHOQOL-Bref physical remains the same and, consequentially, the difference between T0 and T8 do not reach any statistical significance.

The perceived quality of life in the other domains did not change during the treatment in both groups. Thus no other differences were found between and within groups.

**Discussion and Conclusion:**

The data presented in the previous paper found that physical activity seems a good adjunctive treatment in the long term management of patients with MDD. These new data indicated that physical activity may also improve the perceived physical quality of life. The dimensions related with social functioning, environment and psychical well being seem do not improved, unexpectedly, during the trial. Two objective dimension not strictly related to the depressive symptoms improved: social functioning and Clinical Global Impression, this discrepancy with a subjective and objective dimension of the well being may supported the Goldberg point of view that subjective quality of life in bipolar and unipolar severe depression patients may not accurately reflect objective functional outcome status, potentially due to diminished insight, demoralization, or altered life expectations over time. It may be that physical activity improve the self perception of physical well being. The physical domains of WHOQOL-Bref inquiry about conditions as sleep, pain, energy, body satisfaction that seems frequently problematic also in remission due to the pharmacotherapy and may be risk factor for relapse/recurrence. Thus physical therapy seems to determinate improvement in depressive aspects not frequently responsive to the drug treatment.

## Introduction

About half of patients who receive treatment with antidepressant drugs for major depressive disorder (MDD) do not achieve full remission of symptoms [1].

Despite treating depression effectively in the short-term antidepressant drugs the problem of relapse/recurrence remains unmitted [1]. Treatment of depression by pharmacological means is likely to leave residual symptoms in most patients. Such symptoms produce impairment and are important risk factors for relapse [1].

Researches continue to seek complementary treatments that may improve residual symptoms of depression, safely, and sustain remission. Physical exercise may be a useful resource [2].

Preliminary results of our research [3] found that depressed women on a supervised exercise regimen adjunctive to the standard therapy with antidepressant drugs had significant improvements in their symptoms over the next 32 weeks, while those on medication alone made only modest gains.

The aim of the present study is to present the data of the above mentioned study [3] on the change in quality of life over 32 weeks in depressed women assuming antidepressant drug with or without physical exercise.

Quality of life has become an important outcome criterion for psychiatric interventions [4], particularly in chronic disorders [4]. Patients with either current depressive disorder or depressive symptoms in the absence of disorder tended to have worse physical, social, and role functioning, worse perceived current health, and greater bodily pain than did patients with no chronic conditions [5]. Nevertheless, there are methodological problems in assessing quality of life. There is a possible measurement overlap between quality of life and psychopathology, especially depression, which may invalidate research results [4]. A recent international study on Longitudinal Investigation of Depression Outcomes (LIDO) shows [6] that measures of physical health, mental health, and quality of life showed consistent longitudinal associations with measures of depressive symptom. Results also show that in the course of MDD measures of mental health changed the most, and physical health the least, infact the measures of change in quality of life were intermediate.

## Methods

###  Study design

Trial with randomized naturalistic controls.

###  Study population

Patients were selected from the clinical activity registries of the Psychiatric Unit of the University of Cagliari, Italy.

The inclusion criteria were the following:

- Female gender

- Age between 40 and 60 years

- Diagnosis of Major Depressive Episode (DSM-IV TR) [4].

- No responsiveness to at least 1 antidepressant at adequate doses (Hamilton Psychiatric Rating Scale for Depression >13 after at least 2 months of pharmacological treatment)

Exclusion criteria were

- Diagnosis of psychotic disorders

- Comorbidity with psychiatric disorders other than Generalized Anxiety Disorder (GAD), Social Phobia (SP), Panic Disorder (PD) with or without Agoraphobia,

- Any contraindications to physical activity

- Diagnosis of neurological and orthopaedic disorders at the time of the study (e.g.: Multiple Sclerosis, Rheumatoid Arthritis, Amyotrophic Lateral Sclerosis, Stroke, radicular or medullary compression, joint fractures, joint surgery, acquired limitations of joint movement).

###  Study protocol

Among a total of 42 eligible patients, 30 (71.4%) consented to participate to the study, and (informed consent was obtained) Patients were randomized after stratification for comorbidity with anxiety disorders (N = 13) between 2 arms of treatment, in a 2:1 fashion.

Group I (cases): 10 patients (4 with comorbidity with anxiety disorder, 1 with SP, 1 PD, 2 GAD) undergoing pharmacological treatment (antidepressants: 6 SSRI, 3 SNRI, 1 NARI) plus physical activity.

Group II (controls): 20 patients (9 with comorbidity with anxiety disorder, 2 with SP, 3 PD, 4 GAD) undergoing only pharmacological therapy (antidepressants: 14 SSRI, 2 SNRI, 1 NARI, SSRI + Tricyclic).

Patients were evaluated at baseline (time 0: before starting the physical activity program), and at 2, 4, 6 and 8 months Diagnoses were carried out according to DSM-IV TR [7] by means of SCID-I (Structured Clinical Interview for DSM-IV Axis I Disorders) [8] administered by trained psychiatrists. The preliminary results already published [3] concerned the comparison between time 0 and time 8 of the data collected by two different trained psychiatric physicians by the following tools: HAM-D (Hamilton Rating Scale for Depression) [9], CGI (Clinical Global Impression) [10] and GAF (Global Assessment of Functioning) [11].

These complementary data regard the comparison of the score of the WHOQOL-Bref [12] in the two groups of patients at the starting time and at the end (32 weeks) of the research.

The WHOQOL-Bref is a relatively new instrument, used to measure quality of life. It is an abbreviated version of the WHOQOL-100 quality of life instrument, developed by the WHOQOL group [13]. The WHOQOL Bref adopts the following definition of health-related quality of life: "the value assigned to duration of life as modified by the impairments, functional states, perceptions, and social opportunities that are influenced by disease, injury, treatment, or policy" [14]. This instrument is being field-tested at present and, according to the WHOQOL group [14] provides an adequate alternative to the WHOQOL-100. It produces scores for four domains related to quality of life (physical health, psychological, social relationships and environment). The Italian version used in the present study has also been reported to have satisfactory psychometric properties [15]. The items are rated on a 5-point Likert scale, reflecting intensity, capacity, frequency or evaluation. The items inquire "how much," "how completely," "how often," "how good" or "how satisfied," with possible answers ranging, from very satisfied to not at all satisfied. The range of scores in each domain is from 4 to 20, where a higher score indicates a better quality of life.

###  Study treatment

The physical activity program included two 60 minute sessions per week, held by a skilled instructor, with ISEF (Physical Education) diploma, Psychology degree and post-degree diploma in Sport Psychopathology (MS).

Each session was set in three steps:

Step I: welcome and warming up (about 5 minutes)

Step II: physiological strengthening (about 50 minutes)

Step III: stretching, cooling down, goodbye (about 5 minutes).

Exercise was performed without machines during step I and III, and with machines during step II, standing in a circle including the instructor, in order to enhance social communication among the members of the working-group. The second step (physiological strengthening) consisted of cardio-fitness machines with different phases: first, every patient could choose a machine and during the exercise she could communicate with the others near or in front of her. After each 4 minute phase, they could rotate to another fitness machine. There were 20 different fitness machines with exercises for arms, legs, and postural muscle strengthening.

Each session was overseen by a physician and a psychologist.

## Results

Table 1 presents the difference among the four WHOQOL-Bref domains scores in the 2 group from t0 and t8 and the comparison between groups at the starting time and at the end of the treatment (32 weeks), and within groups at starting time versus the end of the treatment.

**Table 1:** Comparison of WHOQOL-Bref score subscales (mean ± SD)

	T0 cases (N = 10)	T8 cases (N = 10)	ANOVA 1 way t0 vs t8 cases DF 1, 18, 19	T0 controls (N = 20)	T8 controls (N = 20)	ANOVA 1 way T0 vs t8 controls DF 1, 38,39
WHOQOL-Bref Pych	10.8 ± 1.3	11.1 ± 1.8	F = 0.18 P = 0.674	11.7 ± 1.6	12.0 ± 1.9	F = 0.29 P0.592
ANOVA 1 way cases vs controls DF 1, 28,29				F = 2.37 P = 0.135	F = 1.55 P = 0.224	
WHOQOL-Bref Social relationships	11.2 ± 1.4	11.0 ± 3.9	F = 0.02 P = 0.880	11.1 ± 3.6	10.9 ± 3.7	F = 0.03 P = 0.863
ANOVA 1 way cases vs contols DF 1, 28,29				F = 0.01 P = 0.934	F = 0.03 P = 0.863	
WHOQOL-Bref environment	12.1 ± 2.3	11.5 ± 2.0	F = 0.39 P = 0.541	10.7 ± 2.6	10.1 ± 2.5	F = 0.55 P = 0.461
ANOVA 1 way cases vs controls DF 1, 28,29				F = 2.08 P = 0.161	F = 2.36 P = 0.135	
WHOQOL-Bref Physical health	10.7 ± 2.3	12.9 ± 1.8	F = 5.67 P < 0.03	10.5 ± 3.3	10.5 ± 3.2	F = 0 P = !
ANOVA 1 way cases vs controls DF 1, 28,29				F = 0,03 P = 0.865	F = 4,81 P < 0,05	

The patients in the exercise group had their WHOQOL-Bref physical score improved from T0 to T8, a statistically significant difference. In the control group WHOQOL-Bref physical score from T0 to T8 did not reach statistical significance.

The perceived quality of life in the other domains did not change during the treatment in both groups. Thus no other differences were found between and within groups.

## Discussion

The data presented in the previous paper concerning the same research [3] found that patients with treatment resistant depression using physical activity as adjunctive treatment show a depressive (HAM-D) and general psychopathological (CGI) improvement after long term (32 weeks) physical activity. The global functionality as measured by the GAF also improved. Controls with only pharmacotherapy did not improve their HAM-D, CGI, GAF scores during the 8 months of the trial. Thus physical activity seems a good adjunctive treatment in the long term management of patients with MDD. These new data indicate that physical activity may also improved the perceived quality of life, but only in the "physical domain". Surprisingly, the social relationship, environmental and psychic well being were not improved during the trial. Goldberg recently stated that subjective quality of life in bipolar and severe unipolar depression may not accurately reflect objective functional outcome, possibly due to diminished insight, demoralization, or altered life expectations over time [16]. As presented in the previous paper, two objective dimensions not strictly related to the depressive symptoms improved: social functioning measured by GAF and Clinical Global Impression measured by CGI. Thus, this discrepancy between subjective and objective dimensions of well being may be supported by the Goldberg Hypothesis. From this perspective, it may be hypothesized that physical activity itself may improve one's selfknowledge of physical well being.

The physical domains of WHOQOL-Bref inquiry such as sleep, pain, energy, and body satisfaction (that are problematic aspects but frequently in remission while on pharmacotherapy), may be risk factors for relapse/recurrence [1]. A recent international study on the longitudinal course of depression [1] found that mental health variables all showed large changes during the follow-up of depressed patient, the physical health variables a smaller amount of change, and the quality of life variables were intermediate. Since all persons were initially depressed and most improved, authors say that it is likely that improvement in the underlying depression caused the changes in the other dimensions, and that this improvement had the most impact on mental health, the least on physical health, and intermediate impact on quality of life.

In our study physical activity as an adjunctive treatment may play a role in the longterm improvement of depressive symptoms, objective general clinical well being and social functioning and subjective perception of physical well being.

These new data confirm that exercise seems to interact with other dimensions of well being in depressed women, and not merely with their mood.

There are probably three different factors determining the efficacy of group exercise in the treatment of depression: 1) enhancing social communication among the members of the working-group may improve the tendency to interpersonal difficulties in this population. This is a nonspecific factor and may be similar to other social activities [17] The remaining two are more exercise specific; 2) the production of biological modifications [18-23]: a) improving elevation of beta endorphins in some areas of the central nervous system. b) a decrease in the level of cortisol in the blood; c) the production of serotonin in the central nervous system; d) elevation of body temperature, leading to relaxation 3) enhancement of social communication secondary to the improvement of "biological" background (endorphins, serotonin, cortisol).

Concerning these effects (particularly 2 and 3) it is interesting to point out that according to the latest theories [24] these interact with the mechanism of stress. Stress may be caused by any environmental change, whether internal or external, that disrupts the maintenance of homeostasis, and initiates a series of neuronal responses to prepare the organism to adapt to this new environmental challenge. Stress can be the most significant causal agent of depression, together with genetic vulnerability. Corticotrope and serotonin systems, that can be influenced by exercise, have largely been involved in depressive symptoms and antidepressant therapies are known to act through them. Stress-induced decrease in hippocampal neurogenesis might be an important feature associated with depressive episodes. Both corticotrope and serotonin systems induce sustained modifications in adult hippocampal neurogenesis and neurons in the hippocampal formation are among the most sensitive to the deleterious effects of stress. Recently was found that, many, but not all of the beneficial effects of exercise on brain function depend on increasing hippocampal neurogenesis, including improved cognition and reduced anxiety [25].

## Study limitations

Small sample size limited to adult females. The control group was not subjected to structured rehabilitation activity or placebo, or with exercise conducted alone, so it was impossible to evaluate whether the improvement was due to a non specific therapeutic effect associated with taking part in a social activity.

## Conclusion

Long term use of exercise provides a better subjective physical quality of life for MDD patients. Randomized controlled trials with a placebo group and a large sample size are needed to confirm the results.

## Competing interests

The author(s) declare that they have no competing interests.

## Authors' contributions

MGC conceived of the study, participated in the design of the study, coordinated the study and performed the statistical analysis. MCH, AP, MS, ALF, FAM, AC participated in the coordination of the study and carried out the research. MGC, MCH, AB and MS drafted the manuscript. MS also conceived of the study. All authors read and approved the final manuscript.

## References

[B1] Fava GA Ruini C Long-Term treatment of depression: there is more than drugs Recent Prog Med 2002 93 6 343 5 12085711

[B2] Trivedi MH Greer TL Grannemann BD Chambliss HO Jordan AN Exercise as an augmentation strategy for treatment of major depression J Psychiatr Pract 2006 12 205 13 10.1097/00131746-200607000-00002 16883145

[B3] Pilu A Sorba M Hardoy MC Floris AL Mannu FA Seruis ML Velluti C Carpiniello B Salvi M Carta MG Efficacy of physical activity in the adjunctive treatment of major depressive disorders: preliminary results Clinical Practice and Epidemiology in Mental Health 2007 3 8 10.1186/1745-0179-3-8 PMC1976311 17620123

[B4] Aigner M Förster-Streffleur S Prause W Freidl M Weiss M Bach M What does the WHOQOL-Bref measure? Measurement overlap between quality of life and depressive symptomatology in chronic somatoform pain disorder Soc Psychiatry Psychiatr Epidemiol 2006 41 1 81 6 10.1007/s00127-005-0997-8 16341612

[B5] Hays RD Wells KB Sherbourne CD Rogers W Spritzer K Functioning and well-being outcomes of patients with depression compared with chronic general medical illnesses Arch Gen Psychiatry 1995 52 11 19 7811158 10.1001/archpsyc.1995.03950130011002

[B6] Diehr PH Derleth AM McKenna SP Martin ML Bushnell DM Simon G Patrick DL Synchrony of change in depressive symptoms, health status, and quality of life in persons with clinical depression: Health and Quality of Life Outcomes 2006 4 27 16638129 10.1186/1477-7525-4-27 PMC1524937

[B7] American Psychiatric Association Diagnostic and statistical manual of mental disorders, 4th edition, text revision (DSM-IV TR) 2000 Washington DC; American Psychiatric Press

[B8] First MB Spitzer RL Gibbon M Williams JBW Structured Clinical Interview for DSM-IV-TR Axis I Disorders, Research Version, Patient Edition (SCID-I/P) 2002 New York: Biometrics Research, New York State Psychiatric Institute

[B9] Hamilton M A rating scale for depression J Neurol Neurosurg Psychiat 1960 23 56 62 14399272 10.1136/jnnp.23.1.56 PMC495331

[B10] Guy W Clinical Global Impression ECDEU Assessment Manual for Psychopharmacology, revised 1976 Rockville MD: US Department of Health Education and Welfare 217 222

[B11] American Psychiatric Association Global Assessment of Functioning Scale (GAF) Asse V. American Psychiatric Association. Diagnostic and Statistical manual for Mental Disorders. (DSM IV) 1994 Fourth Washington DC; American Psychiatric Press

[B12] World Health Organization WHOQOL-Bref: Introduction, administration, scoring and assessment of the generic version. field trial version 1996 Geneva: World Health Organization

[B13] World Health Organization The World Health Organization Quality of Life assessment (WHOQOL) position paper from the World Health Organization Soc Sci Med 1995 1403 1409 8560308 10.1016/0277-9536(95)00112-k

[B14] World Health Organization Development of the World Health Organization WHOQOL-Bref Quality of Life Assessment The WHOQOL Group Psychol Med 1998 28 551 558 10.1017/s0033291798006667 9626712

[B15] De Girolamo G Rucci P Scocco P Becchi A Coppa F D'Addario A Daru E De Leo D Galassi L Mangelli L Marson C Neri G Soldani L Quality of life assessment: validation of the Italian version of the WHOQOL-Bref Epidemiol Psichiatr Soc 2000 9 45 55 10859875 10.1017/s1121189x00007740

[B16] Goldberg JF Harrow M Subjective life satisfaction and objective functional outcome in bipolar and unipolar mood disorders: a longitudinal analysis J Affect Disord 2005 89 1–3 79 89 10.1016/j.jad.2005.08.008 16249035

[B17] Moos RH Cronkite RC Symptom-based predictors of a 10-year chronic course of treated depression J Nerv Ment Dis 1999 187 6 360 368 10.1097/00005053-199906000-00005 10379723

[B18] Sarbadhikari S Saha A Moderate exercise and chronic stress produce counteractive effects on different areas of the brain by acting through various neurotransmitter receptor subtypes: a hypothesis Theor Biol Med Model 2006 23 3 33 10.1186/1742-4682-3-33 PMC1592480 16995950

[B19] Lobstein DD Ismael AH Rasmussen CL Decreases in resting plasma beta-endorphin and depression scores after endurance training J Sports Med Phys Fitness 1991 31 4 543 551 1806732

[B20] Blumenthal JA Babyak MA Moore KA Craighead WE Herman S Khatri P Waugh R Napolitano MA Forman LM Appelbaum M Doraiswamy PM Krishnan KR Effects of Exercise Training on older Patient With Major Depression Arch Intern Med 1999 159 2349 2356 10.1001/archinte.159.19.2349 10547175

[B21] Brosse AL Sheets ES Lett HS Blumenthal JA Exercise and the treatment of clinical depression in adults: recent findings and future directions Sports Med 2002 32 741 760 10.2165/00007256-200232120-00001 12238939

[B22] Gullete ED Blumenthal JA Exercise Theraphy for the prevention and Treatment of depression J Prac Psych And Behav Hlth 1999 5 263 271

[B23] Pennix BH Rejeski WJ Pandya J Exercise and depression symptoms: A comparison of Aerobic and Resistance Exercise Effects on Emotional and Physical Function in Older Persons With High and Low Depressive Syntomatology Journal of Gerontology: Psychological Sciences 57B, No 2 124 132 10.1093/geronb/57.2.p124 11867660

[B24] Paizanis E Hamon M Lanfumey L Hippocampal neurogenesis, depressive disorders, and antidepressant therapy Neural Plast 2007 737 754 10.1155/2007/73754 PMC1906869 17641737

[B25] Trejo JL Llorens-Martín MV Torres-Alemán I The effects of exercise on spatial learning and anxiety-like behavior are mediated by an IGF-I-dependent mechanism related to hippocampal neurogenesis Mol Cell Neurosci 2007 5 10.1016/j.mcn.2007.10.016 18086533

